# Supplementary material for: Amazonian Bacteria from River Sediments as a Biocontrol Solution against Ralstonia solanacearum
Source: Microorganisms. 2024 Jul 3;12(7):1364. doi: 10.3390/microorganisms12071364 (PMC11278729; doi:10.3390/microorganisms12071364)
Supplement: Supplementary file 1 [file microorganisms-12-01364-s001.zip › microorganisms-3038737-supplementary.pdf]

microorganisms

MDPI

Article

Amazonian bacteria from river sediments as a biocontrol solution against *Ralstonia solanacearum*

Jennifer Salgado da Fonseca [1], Thiago Fernandes Sousa [1], Suene Vanessa Reis de Almeida [2], Carina Nascimento Silva [2], Gleucinei dos Santos Castro [3], Michel Eduardo Beleza Yamagishi [4], Hector Henrique Ferreira Koolen [3], Rogério Eiji Hanada[2], Gilvan Ferreira da Silva [5,*]

Supplementary material

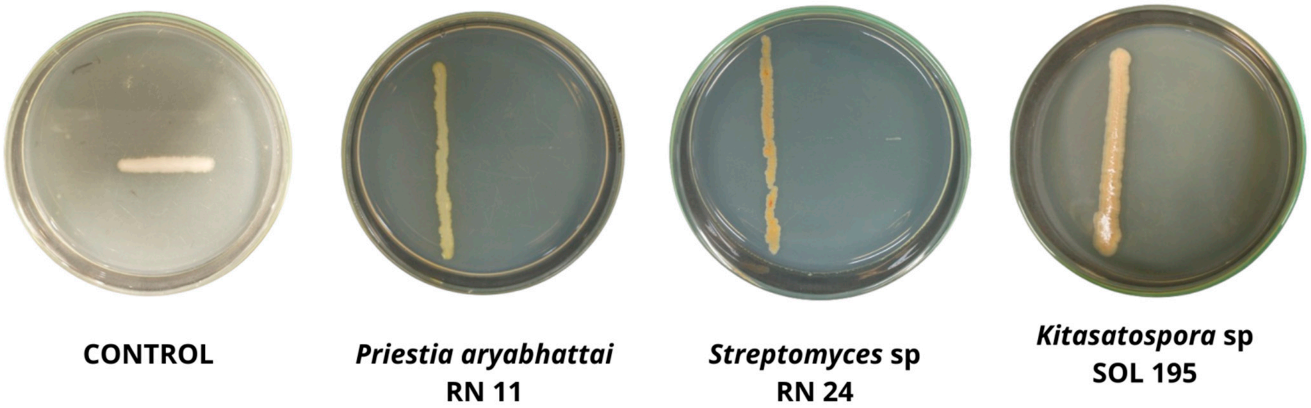

Figure S1. Antimicrobial assay against *Ralstonia solanacerum* using the bacterial strains *Priestia aryabhattai* RN 11, *Streptomyces* sp. RN 24, and *Kitasatospora* sp. SOL 195.

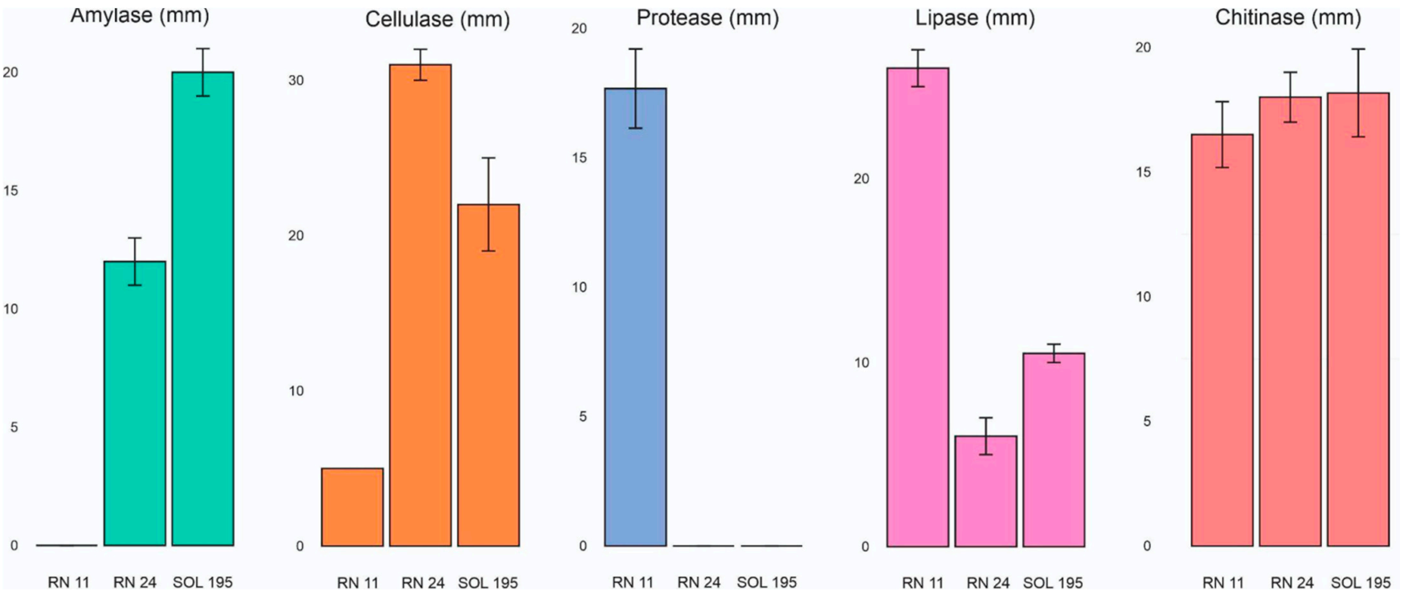

Figure S2. Comparative production of enzymes Amylase, Cellulase, Protease, Lipase, and Chitinase by the bacterial strains *Priestia aryabhattai* RN 11, *Streptomyces* sp. RN 24, and *Kitasatospora* sp. SOL 195.

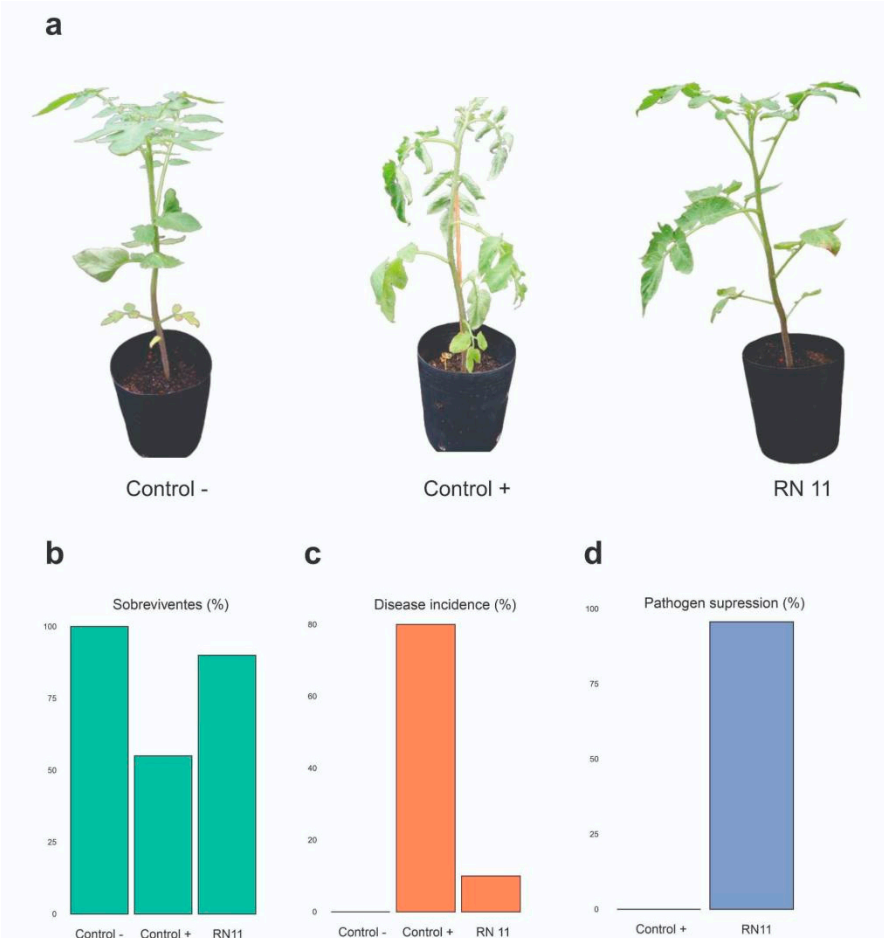

Figure S3. (a) Appearance of seedlings from control groups and those treated with *Priestia aryabhattai* RN 11. (b) Survival indices, (c) disease incidence, and (d) suppression of *Ralstonia solanacearum* in the soil using *Priestia aryabhattai* RN 11 as a biological control agent during the rainy period.

Table S1. Origin of each isolate and Its PASDAAS (percent area specific differential antibiotic activity score) against *Raltonia solanacearum* under *in vitro* condition

Isolates	Geographic coordinates	City	PASDAAS (%)	Type of bacteria
SOL 92	3°21'31.5"S 68°11'59.8"W	Amaturá	21.31±1.03	Actinobacteria
SOL 105	3°51'58.1"S 63°49'03.9"W	Coari	19.15±2.07	Non-filamentous
SOL 134	3°54'56.1"S 62°38'44.2"W		10.59±0.65	Non-filamentous
SOL 229	4°04'21.9"S 63°08'18.7"W		0	Non-filamentous
SOL 65	3°50'05.2"S 62°04'05.2"W	Codajás	16.45±1.26	Actinobacteria
SOL 152	3°16'21.0"S 60°15'01.7"W	Iranduba	12.51±1.78	Non-filamentous
SOL 49	2°34'23.5"S 67°16'08.5"W	Jutaí	11.28±1.42	Actinobacteria
SOL 156	2°46'51.9"S 66°57'27.0"W		15.37±2.51	Non-filamentous
SOL 171			10.54±0.46	Non-filamentous
SOL 230	2°31'19.1"S 66°23'35.0"W		14.37±2.51	Actinobacteria
SOL 110	3°39'59.8"S 64°12'38.2"W	Tefé	0	Non-filamentous
SOL 116			67±2.04	Actinobacteria
SOL 126	3°24'48.9"S 64°32'05.4"W		21.04±1.58	Actinobacteria
SOL 53	2°51'56.9"S 67°46'30.4"W	Tonantins	17.76±2.48	Actinobacteria
SOL 194	2°51'33.7"S 65°11'39.6"W	Uarini	15.67±1.46	Actinobacteria
SOL 195	2°59'08.8"S 65°07'55.2"W		100	Actinobacteria
SOL 196			8.12±0.24	Non-filamentous
SOL 199			10.34±1.53	Actinobacteria
RN 1	3°09'13"S 59°54'42"W	Manaus	13.2±0.82	Non-filamentous
RN 2			0	Non-filamentous
RN 3			16.04±0.81	Non-filamentous
RN 4			14.3±0.26	Non-filamentous
RN 5			9.43±0.41	Non-filamentous
RN 6			8.32±1.14	Non-filamentous
RN 7			7.35±0.92	Non-filamentous
RN 8			13.88±1.59	Actinobacteria
RN 9			10.54±0.46	Actinobacteria
RN 10			9.09±0.98	Non-filamentous
RN11			100	Non-filamentous
RN 12			0	Non-filamentous
RN 14			29.27±0.79	Non-filamentous
RN 15			21.94±1.68	Non-filamentous
RN 16			10.8±0.74	Non-filamentous
RN 22			36±1.37	Non-filamentous
RN 23			7.28±1.22	Non-filamentous
RN 24			87.55±0.8	Actinobacteria
RN 32			11.13±0.92	Actinobacteria
